# Effect of Drought on Bean Yield Is Mediated by Intraspecific Variation in Crop Mixtures

**DOI:** 10.3389/fpls.2022.813417

**Published:** 2022-01-27

**Authors:** Akanksha Singh, Inea Lehner, Christian Schöb

**Affiliations:** Agricultural Ecology Group, Institute of Agricultural Sciences, ETH Zurich, Zurich, Switzerland

**Keywords:** intraspecific variation, common bean, drought stress, intercropping, crop diversity, plant interactions

## Abstract

Increasing plant diversity in agricultural systems provides promising solutions for sustainably increasing crop yield. It remains unclear; however, how plant–plant interactions in diverse systems are mediated by plant genetic variation. We conducted a greenhouse experiment in which we grew three varieties of common beans with three companion plant species (chickpeas, sorghum, and sunflower) in different combinations (crop mixtures, bean cultivar mixtures, and monocultures), with and without drought stress. We hypothesized that under drought stress, the effect of companion plant species on bean yield would be mediated by the drought tolerance potential of the species. We further hypothesized that this effect would vary across different bean cultivars. Overall, we show that the effect of companion plant species on bean yield was not influenced by drought stress; instead, it was dependent on the identity of the bean variety. This could partially be explained by variation in growth rate between bean varieties, where the fastest growing variety recorded the highest yield increase in plant mixtures. The effect of companion plant species on chickpea biomass, however, was potentially influenced by chickpea drought tolerance potential; chickpea biomass was recorded to be higher in plant mixtures than in its monoculture under drought conditions. Our study highlights that to develop plant mixtures, it is not only important to consider the functional traits of the interacting plant species, but also those of the different plant varieties. We further suggest that stress tolerance can be a useful trait for initial selection of plant varieties when developing crop mixtures.

## Introduction

Agricultural intensification for increased production is one of the main causes of biodiversity loss worldwide (Donald et al., 2006; van Lexmond et al., 2015). Enhancing crop diversity in agricultural systems proposes a promising solution to promote biodiversity and the associated ecosystem services. One practice of enhancing crop diversity in the field, specifically in the tropics, is intercropping (Vandermeer, 1992). Intercropping involves growing two or more crop cultivars or species that coexist for a period of time, on the same piece of land (Brooker et al., 2015). In recent decades, a wide array of research has been conducted to determine optimal crop mixtures that result in higher productivity from the available land (Vandermeer, 1992; Brooker et al., 2015). However, most of the previous work has focused on interspecific plant interactions in determining such productive mixtures. The role of intraspecific plant variation in mediating these plant–plant interactions is often unexplored (Schöb et al., 2015; Haug et al., 2021), particularly under environmental stress.

Crop production can be limited by various abiotic stress factors, such as the availability of light or water (Marschner, 2012). With a changing climate, heat and drought stress are predicted to be among the main challenges for crop production, particularly in areas already exposed to high temperatures and water scarcity (Fereres and Soriano, 2006; Hatfield et al., 2011). Developing crop mixtures that promote complementarity effects between plants can assist in mitigating these challenges of crop production. Both facilitative interactions and niche differentiation lead to complementarity effects between plant species. Facilitative interactions are positive interactions that occur between physiologically independent plants (Brooker et al., 2008). Niche differentiation occurs among plant species when they have different resource requirements or vary spatially and/or temporally in their resource/habitat use (Tilman et al., 2001). A common example of a crop mixture that promotes complementarity effects between plants to combat water stress is when a C_4_ crop is mixed with a C_3_ crop. C_4_ crops are known to have enhanced water use efficiency (Vogan and Sage, 2011); hence, they potentially compete less for available water resources. It is important to note, however, that the direction or the strength of such beneficial plant–plant interactions is context dependent and can vary with the environmental conditions in which they occur. Thus, when developing crop mixtures, it is crucial to test their productivity under relevant stress factors.

The influence of environmental context on net plant–plant interactions has been widely explored under the “stress gradient hypothesis” (SGH). The SGH predicts that the frequency of net positive plant–plant interactions are more common under conditions of high abiotic stress than under low or no stress (Maestre et al., 2009). However, the impact of environmental stressors on plant interactions can vary with multiple factors, such as the type and gradient of the stressors or the competitive ability of the species involved (Maestre et al., 2006; Soliveres et al., 2015). Such understanding is specifically relevant for developing intercropping systems. For example, maize and common bean are traditionally grown in crop mixtures in Latin America and Africa (Vandermeer, 1992; Nassary et al., 2020). Maize benefits from the nitrogen provided by bean plants and beans may benefit from the weed reduction services provided by maize plants, potentially leading to a net positive interaction between the two crops. However, both maize and common bean have a low drought tolerance and drought is one of the main limiting factors for their production (Beebe et al., 2014; Sah et al., 2020). Hence, when grown together under water deficit conditions, their net positive interaction may reduce or even shift to competition for both species. Similar to such trends observed for plant–plant interactions, plant genetic variation could also influence the impact of environmental stressors on plant interactions.

In intensively cultivated fields, typically only a single cultivar of a specific crop species is grown. The cultivars used are usually suited for high input systems and are bred in monocultures for one specific trait, e.g., high productivity (Hoisington et al., 1999; van de Wouw et al., 2010; Murphy et al., 2013). The different commercially available cultivars have a varying range of tolerance to a specific stressor. Hence, they could also vary in how they interact with the neighboring plant species in crop mixtures (Litrico and Violle, 2015). This further suggests that if the strength or the direction of plant–plant interactions varies across the gradient of environmental stress, this may not only be mediated by the functional traits of the interacting species, but also by the properties of the selected cultivar.

Common bean (*Phaseolus vulgaris* L.) is considered the most important grain legume, supporting food security and human nutrition globally. There is wide genetic variability in common beans considering the shape, size, and color of their grains, which leads to a large number of distinct commercial classes (Pereira et al., 2019). Drought is one of the most common reasons for yield loss in common beans (Beebe et al., 2013, 2014). Yet, approximately 60% of bean production occurs in areas that are prone to water deficiency and where unexpected drought results in up to 80% of yield loss (Acosta Gallegos and Shibata, 1989; Broughton et al., 2003; Rosales et al., 2012). This study is part of the “DiverBeans” project that aims to improve bean production in North Macedonia, a country where a vast majority of cropland is rain-fed and only approximately 10% is irrigated (Aksoy et al., 2020). Total bean production has reduced by about 30% in the last 10 years (FAO, 2021). In a previous stakeholder workshop, we determined water limitation and sun intensity to be the main limiting factors for bean production (Singh et al., 2019). One solution we proposed to counteract these challenges was to increase diversity on bean fields. We conducted this study to select bean varieties and crop mixtures that can be trialed in farmer fields in North Macedonia.

First, we conducted a preliminary greenhouse trial at ETH Zürich in which we screened 13 varieties of common bean and selected three with the highest drought tolerance index (i.e., the variety that had the highest yield under drought conditions relative to other varieties; Supplementary File 1: Table S3). In the second greenhouse experiment, the chosen varieties were mixed with three companion plant species (sunflower, chickpeas, and sorghum) in different crop and bean cultivar mixtures. We predicted the additional species to vary in their range of drought tolerance, with sorghum being the most tolerant as it is a C_4_ crop (Varoquaux et al., 2019), followed by chickpeas and, with sunflower being the least tolerant as it is a water demanding crop (Hussain et al., 2018). We hypothesized that the interaction of beans with the additional crop species will be influenced by water stress, by identity of the bean varieties, and by the drought stress tolerance of the additional species.

## Materials and Methods

### Experimental Design

We used four plant species in total: common bean (*P. vulgaris* L.), chickpea (*Cicer arietnum* L.), sorghum [*Sorghum bicolor* (L.) Moench], and sunflower (*Helianthus annus* L.). For beans, we used three different varieties called “*Borlotti Sasso Rosso*” (Variety 1), “*Borlotti Taylors*” (Variety 2; Bioseme company, Italy), and “*Borlotti Lingua de Fuoco*” (Variety 3; Fratelli Ingegnoli company, Italy).

Sunflower and chickpea seeds were obtained from local farmers in the Mustafino and Gevgelija regions, respectively, of North Macedonia. The sorghum variety used is called “Goldhirse” (Botanik Samereien GmbH, Switzerland). Common beans were inoculated with a dried peat-based inoculant containing *Bradyrhizobium japonicum* bacteria (HISTICK^®^ Soy, BASF, Switzerland) before sowing.

The three bean varieties and the three plant species were grown in different cultivar and crop species mixtures (Monocultures, Cultivar mixtures, 2-species mixtures, and 4-species mixtures). In total, we had 21 different combinations (Table 1). Each of the 21 combinations were subjected to two different water treatments- “no water stress” and “drought stress”. We replicated each of our 42 treatments eight times, giving us 336 pots. Our pots were divided into eight blocks and each block contained one replicate of all the 42 treatments.

**Table 1:** Different plant combinations applied in the experiment.

Monocultures	Cultivar mixtures	2-species mixtures	4-species mixtures
Variety 1	Variety1 + Variety 2	Variety1 + Sorghum	Variety1 + Sunflower + Chickpeas + Sorghum
Variety 2	Variety1 + Variety 3	Variety1 + Sunflower	Variety2 + Sunflower + Chickpeas + Sorghum
Variety 3	Variety3 + Variety 2	Variety1 + Chickpeas	Variety3 + Sunflower + Chickpeas + Sorghum
Sunflower		Variety2 + Sorghum	
Sorghum		Variety2 + Sunflower	
Chickpeas		Variety2 + Chickpeas	
		Variety3 + Sorghum	
		Variety3 + Sunflower	
		Variety3 + Chickpeas	

We followed a substitutive design in our experiment and maintained a density of four plants in all pots. This means that in our *monoculture* pots, we had four plants of the same species or variety; in the *cultivar mixture* pots, we had two plants from each of the two bean varieties; in the *2-species mixture* pots, we had two plants of the same bean variety with two plants of one of the companion plant species; and in the *4-species mixture pots* we had one plant of each of the plant species.

### Experimental Set Up

The experiment was set up on 6th and 7th January 2020. We sowed three seeds per plant per pot (vol: 5 L, dimensions: 18 × 18 cm) in “Rasenerde 146” soil (Ricoter, Switzerland) with 30% sand, 20% coco peat, 20% perlite, and 30% topsoil. We renamed our cultivars as Variety 1 (*Borlotti Sasso Rosso*), Variety 2 (*Borlotti Taylors*’), and Variety 3 (*Borlotti Lingua de Fuoco*). One week after sowing, 2 g of fertilizer (Biosol Universaldünger vegan, Andermatt Biogarted) was added to each pot.

Plants of all four species started to germinate and emerge approxmately 1 week after sowing. Variety 3 showed poor germination rate in our experiment and we did not have sufficient plants from this variety in 38 pots. These pots were removed from the final analysis. Hence, for our treatments including Variety 3, we had five to eight replicates.

Drought stress started on the 5th week after experimental set up. Until then, all plants received equal amounts of water. Bean plants started to develop flowers when they were approximately 5 weeks old. The period between flowering and fruit pod development is when beans are most susceptible to drought (Dipp et al., 2017). Therefore, we started drought stress by the 5th week. Plants without stress were given 90% of soil water holding capacity of water (i.e., water holding capacity of soil in the pot) twice a week (~600 ml). For plants with drought stress treatment, the amount of water was reduced gradually; in the 5th week, these plants were given 60% (~350 ml), in the 6th week 40% (~250 ml); and in the 7th, 8th, and 9th week 20% (~150 ml) soil water holding capacity of water, respectively. By the 9th week, all bean plants had developed matured fruit pods and in the 10th week, all plants were watered equally.

To estimate water holding capacity of soil in the pot, we used three 5 L pots filled with experimental soil and saturated them with water. These pots were left covered for 24 h, after which we determined their weight (pot + wet soil). Then, we placed the pots for 48 h in an oven at 60°C to determine their weight again (pot + dry soil). Water holding capacity of soil in the pot was calculated by subtracting the average weight of the three pots with wet soil with the average weight of pots with dry soil.

The experiment was carried out at Lindau experimental station of ETH Zurich in Eschikon, Switzerland. Greenhouse temperature was maintained at (max:min) 20:16°C, average humidity was 50.1%, and we used 16/8 h (Light/Dark) light cycle throughout the experiment. The photosynthetic photon flux density was approximately 338 μmol/m^2^/s and the light source was from cool-white fluorescent lamps.

### Data Collection

Data were collected on the 6th, 8th, 9th, and 11th week after experimental set up. We collected data on number of leaves, flowers, and fruit pods per pot. By the 6th week, some plants had small emerging fruit pods, but by the 8th and 9th weeks, we observed filled pods. On the 6th (at initiation of drought stress) and 8th week, we also collected fluorescence data from one leaf of bean plants to determine photosynthetic efficiency (using Fluorometer Mini-Pam II, Walz). We have used quantum yield (QY) as a parameter to determine photosynthetic efficiency in our study. QY estimates the efficiency at which light absorbed by photosynthetic system II (PSII) is used for reduction of primary quinone electron acceptor of PSII (Q_A_; Baker, 2008).

In all pots except *4-species mixture* pots, data were collected from two bean plants. For *monoculture*, we collected data from two plants of the same bean variety and for *cultivar mixture* pots, we collected data from one plant of each of the two bean varieties. Data were collected from the same plants per pot during each data collection time point. Due to time constraint, fluorescence data were only collected from Blocks 1, 2, 7, and 8.

After formation of filled pods, bean plants need another 2–3 weeks before the pods dry out and the seeds inside are fully matured. Therefore, after week 9, we left the plants to dry for 2 more weeks for final seed collection. On week 11, we started collecting final yield data from bean plants. We recorded the total number of bean fruit pods per pot from all bean plants and collected the fruit pods in separate paper bags. We also counted the number of seeds within fruit pods for all bean plants. In addition, we collected chickpea, sunflower, and sorghum plants in separate bags. These plants were dried in an oven at 60°C for 4 days to measure their aboveground dry biomass. Bean plant biomass was not measured as the plants completely dried out and dropped their leaves by the time they formed mature fruits.

For our data analysis below, we have focused on seed number and not seed mass. We did this due to the timing of data collection. By the 10th week, our greenhouse facility had to close due to the COVID-19 pandemic and we had to get special permission to finish our experiment. Hence, we had to collect yield data for all bean plants in the same week. All bean plants had developed fully formed pods by then; however, the stage of seed maturation within the pods varied. This meant that all plants had fruit pods with full sized seeds inside, but the seeds were at different stages of maturation (drying). Therefore, seed number is a more standardized variable in our experiment for beans. By the time we collected bean data, the additional crops had not fully matured. Therefore, we only collected their aboveground biomass.

### Data Analysis

The number of plants per pot of a specific plant species varied across different treatments. Additionally, as mentioned above, we could not collect yield data for all plant species. Therefore, our data analysis focuses on two separate plant yield aspects. First is the total yield/biomass of each of the plant species per pot as this is of interest to the farmers. Second is yield/biomass of individual plants of each of the species; this better helps us understand how a specific species interacts with the other species, with or without drought stress.

We ran linear and generalized linear mixed effect models (LMER and GLMER) using the lmer package. When GLMER models were run, we used the “gamma” family of error distribution. Our final models with random effects were selected on the basis of lowest Akaike Information Criterium (AIC) values. To account for spatial variation, block was included as a random effect in all our mixed effect models. We also tested for interactions between the fixed effects in all our models. The data were analyzed using R version 4.0.2 in RStudio version 1.3.1056.

### Common Bean

We analyzed data for the following response variables for beans: seed number per plant, leaf number per plant (in week 6), total bean seed number per pot, quantum yield (QY) at week 6, and QY at week 8.

For the response variables seed number per plant and total seed number, we first ran a LMER model on the whole bean data, including mixture type [crop mixture (i.e., 2-species and 4-species mixtures)/bean cultivar mixture/bean monoculture] and water treatment (drought stress and no water stress) as fixed effects.

To test for interaction effects of variety identity with associated species diversity or with presence/absence of companion plant species on seed number per plant, we split the data and focused on single variety pots (i.e., bean monoculture, 2-species mixtures, and 4-species mixtures). We ran two separate models; in one model, we included variety identity (Variety 1, Variety 2, and Variety 3), presence/absence of companion plant species and water treatment as fixed effects. In the other model, we included variety identity, water treatment, and associated species diversity (0, 1, and 3) as fixed effects. Pots containing a single variety had an associated species diversity of 0 (monoculture), 1 (2-species mixture), or 3 (4-species mixture).

For the response variable leaf number week 6, QY week 6, and QY week 8, we used the single variety data (i.e., bean monoculture, 2-species mixtures, and 4-species mixtures). For these response variables, our models included variety identity and presence/absence of companion plant species as fixed effects. For QY week 8 variable, our model also included water treatment as a fixed effect. We analyzed the data for number of leaves at week 6 to understand the effects of initial plant growth on final bean yield. QY week 6 and QY week 8 data reflect photosynthetic efficiency of bean plants before and after drought stress, respectively.

We also calculated a relative interaction index (RII) for beans (Armas et al., 2004) with single variety data, to compare the relative performance of bean plants when grown with and without companion plant species. We calculated this for each bean variety separately, accounting for the water treatment and spatial variation (Block) by using the formula below:


$$RII=\left(P_{CC}-P_{B}\right)/\left(P_{CC}+P_{B}\right)$$

In the formula above, P_B_ is the yield of a specific bean variety in its monoculture and P_CC_ is the yield of that variety when grown together with a specific companion plant. RII ranges from −1 to +1; a negative RII value indicates that a plant performs better in its monoculture compared to in mixtures, whereas a positive RII indicates better performance in mixtures (Armas et al., 2004). For RII as a response variable, we ran linear models and included variety identity and associated plant identity as fixed effects.

### Companion Plant Species

For the companion plants, we analyzed the data for the variables: total sunflower/sorghum/chickpea biomass per pot and sunflower/sorghum/chickpea biomass per plant per pot. We ran separate models for each of the companion plant species and for each only included data where the respective species were present. For each of the species, we first ran a LMER or GLMER model, including mixture type (crop mixture/monoculture) and water treatment as fixed effects and block as a random effect. To look at the effect of bean variety identity on the companion plant species, we focused on biomass per plant. We included variety identity, associated species diversity (1 and 3), and water treatment as fixed effects.

## Results

### Effect of Mixture Type and Water Treatment

#### Total Yield (Seed Number of Beans) and Total Biomass (Chickpeas, Sunflower, and Sorghum)

Mixture type (crop mixture/bean cultivar mixture/monoculture) affected total yield/biomass for all species (*p* < 0.05; Supplementary File 2: Table S1) and yield/biomass of a specific species was recorded to be the highest in their respective monocultures (Supplementary File 2: Table S2). This is expected because “monoculture” pots had four plants of a specific plant species, and “crop mixture” pots had one or two plants of a specific plant species. The mixture type effect for beans was driven by “crop mixture” (Crop mixture–Cultivar mixture: *t* = 2.19; Crop mixture–Monoculture: *t* = 2.12) and the groups “cultivar mixture” and “monoculture” did not differ significantly from one another (*t* = 0.06, *p* = 0.99). Among all plant species, sorghum recorded the highest decrease in biomass in crop mixtures and total sorghum biomass reduced by ~85% in crop mixture treatment (Supplementary File 2: Table S2).

Total yield/biomass was also affected by water treatment for all species (Supplementary File 2: Table S1); it was higher under no water stress than under drought stress (Supplementary File 2: Table S2). Overall, total yield/biomass of beans (57.4%) and sunflower (55.4%) decreased the most under drought stress in comparison with that of sorghum (33%) and chickpeas (29.7%).

#### Yield (Seed Number of Beans) and Biomass (Chickpeas, Sunflower, and Sorghum) Per Plant

**Figure 1:**
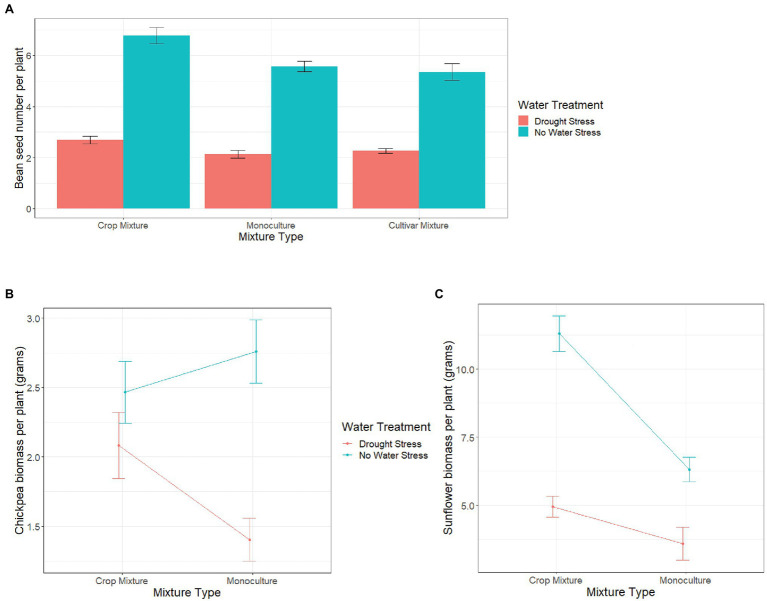
Variation in **(A)** bean seed number per plant, **(B)** chickpea biomass per plant and, and **(C)** sunflower biomass per plant, in different mixture types across two different water treatments (drought stress and no water stress). Error bars represent ±1 SE.

Mixture type significantly affected yield/biomass per plant of all species; however, the effect of a specific “mixture type” varied for the four species (Table 2). For sorghum, biomass per plant was higher in monocultures (4.21 ± 0.06) than in crop mixtures (1.56 ± 0.03), whereas for beans, yield per plant was higher in crop mixtures than in monocultures (Figure 1A). For both sunflower and chickpea, we found biomass per plant to be significantly affected by the interaction between mixture type and water treatment (Table 2). Under “no water stress,” chickpea biomass per plant was higher in monocultures than in crop mixtures, whereas, under drought stress, we recorded an opposite trend (Figure 1B). Although there was no significant interaction, we observed the effect of mixture type on bean yield or on chickpea biomass to vary across bean varieties, under no stress (Supplementary File 2: Figures S1a,b). Finally, sunflower biomass per plant was higher in crop mixture than in monoculture, although the difference between the two mixture types was greater under no water stress (Figure 1C).

**Table 2:** Effect of mixture type (crop mixture/bean cultivar mixture/monoculture) and water treatment on yield (bean) and biomass (chickpeas, sorghum, and sunflower) per plant.

	Explanatory variables		
	Mixture type	Water treatment	Mixture type*water treatment
Bean seed number	MS = 1.13, NumDf = 2, DenDf = 14.83, *F* = 0.62, *p* = 0.549	MS = 559.49, NumDf = 1, DenDf = 229.47, *F* = 309.8, *p* < 0.001	MS =6.21, NumDf = 2, DenDf = 229.42, *F* = 3.44, *p* = 0.033
Sorghum biomass	MS = 96.43, NumDf = 1, DenDf = 93.49, *F* = 80.73, *p* < 0.001	MS = 12.65, NumDf = 2, DenDf = 93.35, *F* = 10.59, *p* = 0.002	–
Chickpea biomass	*X*^2^ = 0.06, deviance = 322.93, Df = 1, *p* = 0.81	*X*^2^ = 4.64, deviance = 322.93, Df = 1, *p* = 0.031	*X*^2^ = 3.49, deviance = 319.44, Df = 1, *p* = 0.061
Sunflower biomass	MS = 8.42, NumDf = 1, DenDf = 4.90, *F* = 1.41, *p* = 0.290	MS = 320.26, NumDf = 1, DenDf = 88.14, *F* = 53.41, *p* < 0.001	MS = 54.82, NumDf = 1, DenDf = 88.35, *F* = 9.14, *p* = 0.003

### Effect of Variety Identity and Associated Species Diversity in Single Bean Variety Pots

#### Bean Yield (Seed Number) Per Plant

Variety identity affected bean yield per plant (Table 3); overall pots with variety 1 had the highest (5.17 ± 0.047), and pots with variety 2 had the lowest yield (3.92 ± 0.045). We found a significant interaction in single variety pots between variety identity and sunflower presence/absence (Table 3). Sunflower presence reduced yield in all varieties, whereby the highest reduction was observed for variety 2 (57%). The effect of sorghum presence/absence on bean yield varied across water treatments (Table 3); bean yield increased in sorghum presence, but the increase was higher under no water stress than under water stress. Although there was no significant interaction between sorghum P/A and variety identity, the increase in yield was lowest for variety 2 in sorghum presence under no stress (20.7%; Supplementary File 2: Figure S2). For variety 1 and 3, sorghum presence increased the yield by 40.5% and 43.9%, respectively.

**Table 3:** Effect of interaction between variety identity and species identity on bean seed number per plant (single variety pots).

Explanatory variables	Mean sq	numDF	denDF	*F*-value	*p*-value
Varieties identity	0.348	2	202.01	25.938	<0.001
Water treatment	3.486	1	202.01	519.100	<0.001
Sunflower P/A	1.465	1	202.91	218.129	<0.001
Chickpeas P/A	0.009	1	202.99	1.325	0.251
Sorghum P/A	0.281	1	202.97	41.809	<0.001
Varieties identity*sunflower	0.065	2	202.00	9.677	<0.001
Sorghum*water treatment	0.0786	1	202.00	11.71	0.007

We further found the interaction between variety identity and associated species diversity to marginally vary across water treatment, this was driven by variety 2 (Variety identity*Water treatment*Diversity: *F*_4,190_ = 2.297, *p* = 0.060). Under no water stress, yield of variety 2 was higher in its monoculture than in 4-species mixtures and was only marginally increased in 2-species mixtures, whereas yield of variety 2 under drought stress was similar in its monoculture and 4-species mixtures and even increased in 2-species mixtures (Supplementary File 2: Figure S3). The strong negative effect of sunflower on the yield of variety 2 and the weak positive effect of sorghum on the yield of variety 2 under no water stress, may have led this variety to record the lowest yield in 4-species mixtures.

#### Relative Interaction Intensity

**Figure 2:**
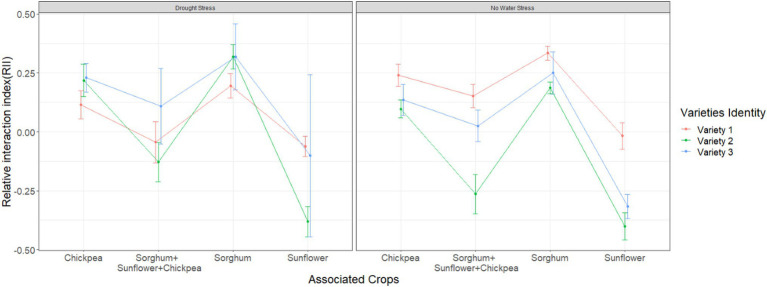
Relative interaction index variation across different varieties in the presence of additional plant species. Error bars represent ±SE.

Relative interaction index was affected by both variety identity (*F*_2,163_ = 9.51, *p* = 0.001) and plant identity (*F*_1,163_ = 43.36, *p* < 0.001). RII was positive for all varieties when they were grown with chickpeas or sorghum and it was the lowest in the presence of sunflower alone, irrespective of drought stress (Figure 2). As mentioned above, different crop species showed varying ranges of tolerance to drought stress in our experiment (in terms of % reduction in biomass under drought stress). This variation was not reflected in RII of beans because we found similar patterns with or without drought stress in the presence of a specific companion plant species. Instead, we found variation across bean varieties in their interaction with companion plant species, across the two water treatments (Figure 2).

Variety 1 performed better in mixtures compared to variety 2 under no drought stress. Furthermore, while we found that yield of variety 1 decreased in sunflower presence, this reduction was the lowest compared to the other varieties. Higher rate of growth may explain the higher yield of variety 1. Therefore, we further investigated the variable bean leaf number at week 6.

#### Leaf Number Week 6

Variety identity had a significant effect on leaf number (*p* < 0.001; Supplementary File 2: Table S3). By week 6, highest leaf number was observed in pots containing variety 1 (35 ± 0.145). Varieties 2 and 3 recorded similar leaf number (variety 2: 29.0 ± 0.140; variety 3: 29.2 ± 0.163). Similar to yield, we found a significant interaction between variety identity and sunflower presence on week 6 leaf number (*p* = 0.011; Supplementary File 2: Table S3). In sunflower presence, leaf number decreased for varieties 2 and 3, but not for variety 1 (Supplementary File 2: Figure S4). We also found a positive correlation between week 6 leaf number and final bean yield per plant (*t* = 11.309, df = 255, *r* = +0.502, *p* < 0.001).

#### Quantum Yield Week 6 and 8

**Figure 3:**
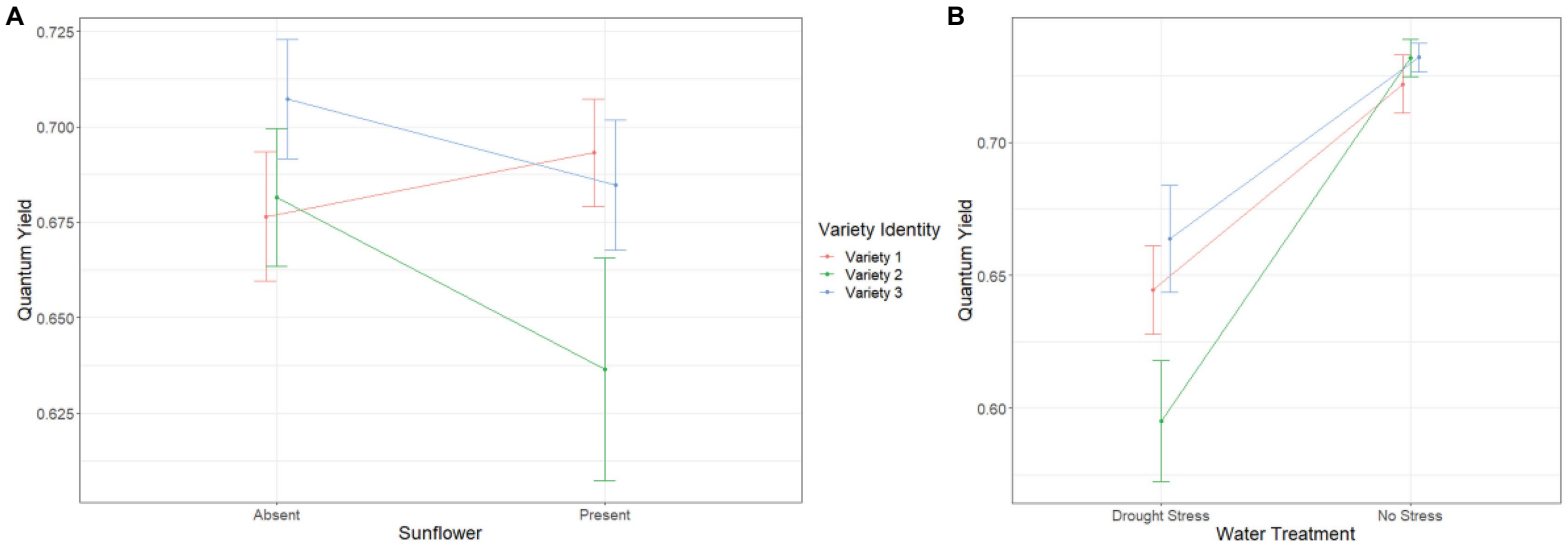
Effect of **(A)** variety identity and sunflower presence, and **(B)** variety identity and drought stress treatment on quantum yield at week 8. Error bars represent ±SE.

We found a significant interaction between variety identity and sunflower presence on QY week 6 (SS = 0.014, *F*_2,100_ = 3.586, *p* = 0.031) and week 8 (SS = 0.022, *F*_2,97_ = 2.675, *p* = 0.063). The pattern of this interaction was similar as for leaf number and similar across the 2 weeks, i.e., before and after drought stress. In sunflower presence, QY decreased for varieties 2 and 3, but not for variety 1 (Figure 3A). Overall, drought stress reduced QY at week 8 for all varieties but the largest reduction was recorded for variety 2 (variety identity*water treatment: SS = 0.028, *F*_2,97_ = 3.363, *p* = 0.038; Figure 3B).

#### Biomass Per Plant of Additional Crop Species

Identity of bean varieties did not influence biomass per plant of any of the additional plant species (Table 4). We found drought stress to significantly reduce biomass per plant of sorghum and sunflower, but not of chickpeas (Table 4). Associated species diversity did not influence sorghum biomass per plant (Table 4) and sorghum biomass was reduced in species mixtures irrespective of diversity. Associated diversity did affect chickpea and sunflower biomass (Table 4) and for both species, biomass per plant was higher when the diversity was 3 than when it was 1.

**Table 4:** Effect of variety identity, water treatment, and associated species diversity on biomass per plant of companion plant species.

	Explanatory variables		
	Variety identity	Water treatment	Associated species diversity
Sorghum biomass	MS = 0.79, NumDf = 2, DenDf = 77.16, *F* = 0.98, *p* = 0.38	MS = 7.77, NumDf = 1, DenDf = 75.66, *F* = 9.62, *p* = 0.002	MS = 0.06, NumDf = 1, DenDf = 75.66, *F* = 0.07, *p* = 0.791
Chickpea biomass	MS = 2.78, NumDf = 2, DenDf = 79.32, *F* = 1.48, *p* = 0.235	MS = 3.20, NumDf = 1, DenDf = 79.29, *F* = 1.70, *p* = 0.196	MS = 9.98, NumDf = 1, DenDf = 79.42, *F* = 5.30, *p* = 0.024
Sunflower biomass	MS = 9.72, NumDf = 2, DenDf = 75.99, *F* = 1.45, *p* = 0.241	MS = 976.98, NumDf = 1, DenDf = 75.37, *F* = 145.69, *p* < 0.001	MS = 418.40, NumDf = 1, DenDf = 75.26, *F* = 62.39, *p* < 0.001

## Discussion

Overall, our study shows that the effect of companion plant species on bean yield was not influenced by drought stress; instead, it was dependent on the identity of the bean varieties. We recorded that sorghum and chickpea presence increased bean yield and sunflower presence reduced bean yield, irrespective of drought stress. Sunflower presence negatively affected bean yield but the bean yield was reduced most for variety 2 and least for variety 1. Sunflower presence further reduced initial growth (leaf number) and photosynthetic efficiency the most for variety 2, but it had no effect on variety 1. Additionally, under drought stress, variety 2 recorded the highest reduction in its photosynthetic efficiency, compared to other varieties. This suggests that the differences observed across varieties in their interaction with the companion plant species was potentially mediated by the variation in their stress tolerance potential and in their competitive ability. Variety 1 was the most competitive and the most stress tolerant, whereas variety 2 was the least competitive and the least stress tolerant variety in our study. Interestingly, even though bean varieties varied in their growth pattern, we found no difference in bean yield between bean monocultures and cultivar mixtures. As we were not able to collect yield or biomass data for all plant species, we focus on yield per plant and have discussed below the implications for the interactions that we observed in our study.

The effect of mixture type on the bean yield and on the biomass of the companion plant species varied for the different species. We suggest that one of the mechanisms for this could be differences in the initial growth pattern of the different species. Both sunflower and beans have a faster vegetative growth rate than sorghum and chickpeas (GRDC, 2017, 2018; NDSU, 2021; Warrick, 2021) and both of these species performed better in mixtures than in their respective monocultures. Sorghum, however, performed better in its monoculture than in crop mixtures, irrespective of the species that it was growing with. It has been shown in several systems that in the absence of nutrients or water limitation, plant species usually compete for light (Aerts, 1999; Craine and Dybzinski, 2013). This can result in faster growing species having a competitive advantage. Due to its relatively slow growth pattern, sorghum potentially competed the least for light. It is possible that the negative effect of beans on sorghum biomass would have been reduced once the bean plants were harvested. Bean is a C_3_ and sorghum a C_4_ crop; a recent meta-analysis comparing C_3_–C_4_ crop mixtures showed that temporal niche differentiation results in significantly higher land equivalent ratios (LER) for such crop mixtures (Yu et al., 2015). As bean yield per plant was recorded to be the highest in 2-species mixtures with sorghum, we suggest bean–sorghum mixture to be one of the mixtures to be trialed in the field. Although, it would be important to test the effect of different temporal configurations (i.e., sowing time) of these crops on their respective yields.

Similar to sorghum, chickpea biomass was also reduced in crop mixtures under no water stress. However, under drought stress, chickpea biomass was recorded to be higher when it was grown in mixtures than in monoculture. Hence, under stress, net facilitation was increased for chickpea plants. This may have occurred because chickpea was potentially the only “drought tolerant” species in our experiment as its biomass was minimally reduced under drought conditions. We only measured biomass of the companion plant species and no other drought response related trait. More data are needed to establish if the increase in chickpea biomass in mixtures under drought stress was really mediated by drought or not. Drought stress reduces leaf size, stem extension, and root proliferation (Farooq et al., 2009). This could have reduced the nutrient and water assimilation potential of bean and sunflower plants (Farooq et al., 2009), further resulting in more resources being available to chickpea plants. As a result of its drought tolerance, perhaps the potential of water/nutrient assimilation was not reduced for chickpea plants under stress. This could have further resulted in chickpea plants competing more for resources with one another when grown in monocultures than in mixtures, under drought conditions.

Irrespective of the effect of our experimental treatments on chickpea plants, bean yield increased in the presence of chickpeas. This suggests that there are additional mechanisms that benefit bean plants when these are grown with chickpeas, e.g., the phosphorous (P) mobilizing potential of chickpea plants (Li et al., 2003). Beans are known to have a low P-uptake efficiency (Föhse et al., 1988), which could have potentially increased in chickpea presence. Root competition could have also affected bean yield positively (Kiær et al., 2013). With faster growth rate, root size of bean plant was potentially larger than of chickpeas’, resulting in their higher assimilation of nutrients. As the productivity per plant of both chickpeas and beans was increased in our study when grown together, this could be a promising mixture for bean farmers in North Macedonia. It is important, however, to first measure the performance of this mixture against pest and pathogen stress. Legumes are usually not grown together because it is assumed that legume–legume mixtures are susceptible to pests and diseases, as the plants are closely related. However, to our knowledge, no study has thoroughly investigated this.

Bean yield significantly reduced under drought stress, irrespective of the companion plant species it was growing with. This may have occurred because the drought intensity in our experiment was too high or because bean plants have a low drought tolerance. It has been suggested that when a limiting resource is the only fundamental abiotic stress factor, facilitation would only occur if the neighboring species increase the availability of this resource (Maestre et al., 2009). When grown with chickpeas and sorghum, nutrients and light were probably not the limiting resources for bean growth as initial growth rate of beans is faster than that of the other species. Therefore, under drought stress, water was potentially the main limiting resource for beans and neither chickpea nor sorghum presence was able to increase its availability.

Our study provides insights for the development of productive crop mixtures for stakeholders by highlighting the role that crop genetic variation can play in mediating plant–plant interactions. It is important to consider though that our study is a pot study, and we could not use the optimum growing conditions for each species inside a greenhouse. Therefore, it would be important to test the selected mixtures in the field. Through our work, we can predict that growing beans with sorghum and chickpeas will potentially increase bean yield, even under drought conditions. However, this would be dependent on the identity of the bean variety used. Farmers can have varying preferences, and, through our study, we provide a varying range of recommendations for the farmers. For example, farmers who prefer sunflower to be the additional crop with beans should choose a faster growing bean variety, such as “variety 1”. Farmers for whom beans are the main cash crop, could grow beans with sorghum or chickpeas. To develop productive crop mixtures, it is not only important to consider the stress tolerant potential of the species involved in the mixtures, but also the stress tolerance variation that exists across cultivars in the different species. We suggest that this is a crucial trait to investigate when breeding plants for crop mixtures.

## Data Availability Statement

The raw data supporting the conclusions of this article will be made available by the authors, without undue reservation.

## Author Contributions

CS procured the funding and originally formulated the idea. AS and CS designed the experiment. AS and IL performed the experiment. AS analyzed the data and wrote the first draft of the manuscript which was commented by CS and IL. All authors read and approved the final version of the manuscript.

## Funding

This study is part of the DiverBean project. The project is supported by the Coop Research Program on “Sustainability in Food Value Chains” of the ETH Zurich World Food System Center and the ETH Zurich Foundation. The Coop Research Program is supported by the Coop Sustainability Fund.

## Conflict of Interest

The authors declare that the research was conducted in the absence of any commercial or financial relationships that could be construed as a potential conflict of interest.

## Publisher’s Note

All claims expressed in this article are solely those of the authors and do not necessarily represent those of their affiliated organizations, or those of the publisher, the editors and the reviewers. Any product that may be evaluated in this article, or claim that may be made by its manufacturer, is not guaranteed or endorsed by the publisher.
