# Supplementary material for: Effect of Drought on Bean Yield Is Mediated by Intraspecific Variation in Crop Mixtures
Source: Front Plant Sci. 2022 Jan 27;13:813417. doi: 10.3389/fpls.2022.813417 (PMC8829134; doi:10.3389/fpls.2022.813417)
Supplement: Supplementary file 1 [file Table_1.docx]

# Supplementary File 1

# Effect of drought on bean yield is mediated by intraspecific variation in crop mixtures

**Akanksha Singh^*^, Inea Lehner, Christian Schöb**

Agricultural Ecology Group, Institute of Agricultural Sciences, ETH Zurich, Zurich, Switzerland

***Correspondence**

Akanksha Singh

email: akanksha.singh@usys.ethz.ch

## Determining drought tolerant bean cultivars

## Summary

We conducted this experiment in the greenhouse to select drought tolerant varieties of common bean (*Phaseolus vulgaris* L.). The aim was to select three bean varieties that could be used for the second plant diversity experiment. The varieties used were sourced from North Macedonia and Italy. We subjected each of the varieties to two different water treatments: ‘No stress’ and ‘Drought stress’. The experiment was terminated in the 8^th^ week when the plants had started to form mature pods. In the 8^th^ week, we collected data on total number and weight of pods per plant, damaged pod number per plant, plant biomass and quantum yield.

Relative to other bean varieties, we found BS1 and BF varieties to have the highest and CW variety to have the lowest drought tolerance. CW is a variety of kidney bean and under drought stress, only one plant of this variety formed pods, resulting in a yield decrease of over 90%. BS1 and BF varieties showed the least reduction in yield (~50%) under drought stress. BS1 further recorded the lowest percentage of damaged pods (~16%) and the highest yield under stress. We also calculated stress tolerant and stress susceptible indices for all varieties; these indices are widely used to screen varieties for their drought tolerance. BS1 and BF varieties ranked the best on these indices and CW variety the worst. For our second experiment, we selected *Borlotti Sasso Rosso* (BS1), *Borlotti Lingua de Fuoco* (BF) and *Borlotti Taylors’* (BT) varieties.

## Methods

### Experimental design

We used 13 varieties of common beans in total for the trial (Table S1). Six out of the 13 varieties were obtained from farmers in North Macedonia. The other seven varieties were bought online from ‘Bioseme’ and ‘Fratelli ingegnoli’ companies in Italy.

Each of the varieties were subjected to two treatments: ‘No stress’ and ‘Drought stress’, resulting in 26 treatments. Each of these treatments were replicated 5-6 times; giving us 146 pots in total. Plants were spread randomly across eight tables within two greenhouses and each of the tables represented a block, hence, we had eight blocks in total.

Table 1: List of common bean varieties used for the trial set up

| **Variety code** | **Type** | **Origin** |
| --- | --- | --- |
| CW | Kidney | Italy |
| KB2 | Kidney | Italy |
| BR | Borlotti | Italy |
| BS1 | Borlotti | Italy |
| BS2 | Borlotti | Italy |
| BT | Borlotti | Italy |
| BF | Borlotti | Italy |
| LJ1 | Borlotti | Macedonia |
| LJ2 | Kidney with white spot | Macedonia |
| LJ3 | Black | Macedonia |
| MJ1 | Borlotti | Macedonia |
| MJ2 | Borlotti | Macedonia |
| MJ3 | Borlotti | Macedonia |

### Study set up

We sowed two seeds per pot (vol: 2.9 litres, dimensions: 16*18 cms.) in ‘Rasenerde 146’ soil bought from Ricoter, Switzerland (sand 30%, coco peat 20%, perlite 20%, country earth 30%). Seeds were coated with *Bradyrhizobium japonicum* bacteria before sowing (HISTICK® Soy, BASF, Switzerland).

Plants started to germinate and emerge approx. one week after sowing and flower buds started to form at 5 weeks after sowing. Drought stress started on the 5^th^ week and until the 5^th^ week, all plants received equal amount of water. By the 5^th^ week, all but two plant varieties (LJ3 and BF) had formed flower buds. Plants without stress were given 90% pot capacity of water twice a week (~400 ml). For plants with drought stress treatment, the amount of water was reduced gradually; in the 5^th^ week these plants were given 60% pot capacity of water (~250 ml), in the 6^th^ week 30% pot capacity of water (~180 ml) and in the 7^th^ week 10% pot capacity of water (~50 ml).

To estimate pot capacity, we used three 2.9-liter pots filled with experimental soil and saturated them with water. These pots were left covered for 24 hours, after which we measured their biomass (Pot+wet soil). Then we placed the pots for 48 hours in an oven at 60 degrees to measure their biomass (Pot+dry soil). Pot capacity was calculated by subtracting the average biomass of the three pots with wet soil with the average biomass of pots with dry soil.

### Data collection

We started collecting data on the 5^th^ week after experimental set up, the day before we started the drought stress treatment. After this weekly data was collected for the following variables per plant: number of flowers, number of leaves and number of pods. Final data collection was carried out on the 8^th^ week after experimental set up. We collected data per plant on number of damaged and undamaged pods, pod weight per plant and Quantum Yield from one leaf (using Fluorometer Mini-Pam II, Walz). Finally, plants were cut from base of the stem, placed in bags and dried in the oven at 70°C for three days to measure their aboveground dry biomass.

### Data analysis

Two plants died during the experiment, one of BS2 and one of CW variety. The BS2 variety plant potentially caught a soil disease and died in the 6^th^ week. The CW variety plant was under drought stress treatment and died due to the drought conditions by the 8^th^ week. Hence, our final sample size was 144 plants.

Our data analysis can be split into two steps, as a first step we analyzed if the variables bean yield, quantum yield and plant biomass were significantly affected by our experimental treatments (bean variety and drought stress). In the second step, we calculated stress tolerant and stress susceptible indices for different varieties (1–3), in order to rank them for their drought tolerance potential.

**Step 1:** To analyze the effects of our treatments on the response variables, we ran linear model or generalized linear models, including ‘plant variety’ and ‘drought stress’ as the main explanatory variables. Data was analyzed for the following response variables: yield per plant (weight of all pods per plant), plant biomass, proportion yield loss under stress, percentage of damaged pod number and Quantum Yield (QY). For the response variable ‘proportion yield loss under water stress’, only data of plants with drought stress was used.

- Proportion yield loss was calculated using the formula:

*yield of plant of variety X under drought stress treatment / Average yield of plants of variety X under no stress treatment*

- Percentage of damaged pod number per plant was calculated using the formula

*(Number of damaged pods of a plant/total number of pods of a plant)*100*

**Step 2:** We picked six indices for our study that are widely used to screen genotypes for drought tolerance. These indices are developed to consider the following factors (4): (a) uniform performance of a genotype under stressed and non-stressed conditions, (b) high performance under stressed conditions, (c) high performance under non-stressed conditions and, (d) low performance in both stressed and non-stressed conditions. The indices are described below:

- Stress tolerance index(4) (STI) = [(Yp x Ys) / Yp^2^]
- Stress susceptibility index(5) (SSI) = [1 - (Ys / Yp)] / [1 - (Ys1 / Yp1)]
- Geometric mean productivity(4) (GMP) = square root(Ys× Yp)
- Yield stability index(6) (YSI) = Ys/Yp
- Yield index(7) (YI) = Ys/ Ys_1_
- Mean productivity(2) (MP)= (Ys+Yp)/2

Where Yp is the average yield of a specific variety under no stress, Ys is the average yield of a specific variety under drought stress, Yp_1_ is the average yield of all plants under no stress and, Ys_1_ is the average yield of all plants under drought stress.

For all these indices except for the stress susceptibility index (SSI), varieties with the highest values were considered drought tolerant, whereas for SSI, varieties with the lowest value were considered tolerant.

## Results

Two of the varieties, LJ3 and BF, matured slower in comparison to other varieties. These varieties started to form pods only in the 7^th^ week, whereas, other varieties started forming pods in the 8^th^ week. Therefore, overall yield (weight of all pods per plants) for these varieties is lesser than for other varieties. Under drought treatment, only one plant of CW variety produced pods, one plant died, and the rest remained at the bud stage.

All our response variables (yield per plant, biomass, Quantum Yield, proportion yield loss and percentage damaged pods number) were significantly affected by our treatments, bean variety and drought stress (Table S2).

Table 2: Effect of main experimental variables on bean yield variables

|  | **Response variables** | | | | |
| --- | --- | --- | --- | --- | --- |
|  | **Plant Biomass** | **Total yield** | **Proportion yield loss** | **% damaged pods** | **Quantum Yield** |
| **Variety** | F12,123=3.36, P<0.001 | F12,123=10.43, P<0.001 | F12,61=3.13, P=0.002 | F12,123=12.738, P<0.001 | F12,123=2.27, P=0.002 |
| **Drought stress** | F1,123=17.13, P<0.001) | F1,123=405.38, P<0.001 | NA | F1,123=19.413, P<0.001 | F1,123=9.36, P=0.003 |

**Total yield:** Overall, plants with drought stress had lower yield (mean weight: 4.71±0.426 gms, mean healthy pod number: 2.51±0.179) than plants without any stress (mean weight: 15.77±0.584 gms, mean healthy pod number: 6.43±0.264). Amongst plants with ‘no stress’, yield was highest for LJ1 (mean weight: 19.06±1.569 gms) and lowest for LJ3 variety (9.77±1.274 gms) of plants. Amongst plants with ‘drought stress’ yield was highest for BS1 (mean weight: 8.42±2.669 gms) and lowest for CW (mean weight: 0.59±0.52 gms) variety. We found no correlation between biomass and yield (t = 1.1033, df = 142, P= 0.2718, r= 0.092).

**Proportion yield loss:** As mentioned above, in all bean varieties yield decreased under drought treatment. However, there was large variation in the proportion of yield decrease across varieties. CW variety recorded the highest proportion of yield decrease under stress (>90%) and BF and BS1 varieties recorded the lowest decrease (~50%, Figure S1).


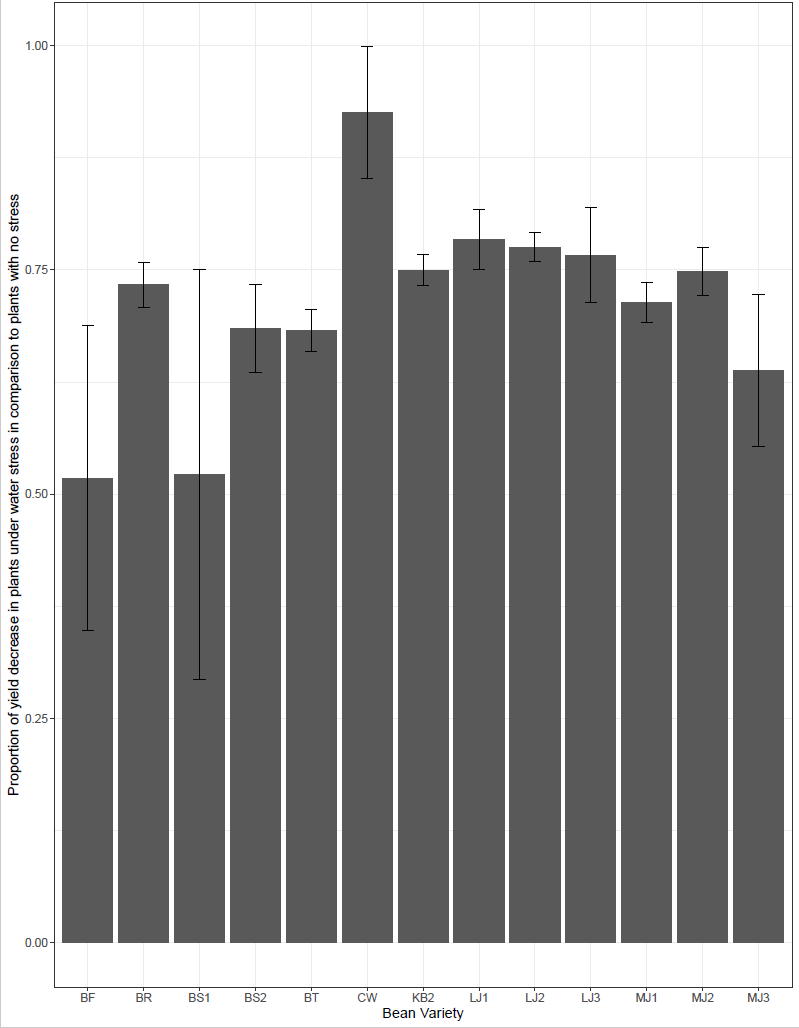


*Figure 1: Proportion of yield decrease in plants under drought stress in comparison to plants without stress across bean varieties*

**Percentage damaged pods:** We introduced drought stress when plants were five weeks old and had reached the flowering stage. Following the drought stress, plants did start to form pod, although majority of the pods in stressed plants did not develop (stayed between 5-10 cms in size) or turned brown. Hence, we found a higher percentage of damaged pods in stressed plants in comparison to non-stressed plants (Figure S2), for all bean varieties. Here again, the BS1 variety had the lowest percentage (~16%) of damaged pods under stress, whereas, KB2 variety had highest (~87%). Only one plant of CW variety produced pods under drought stress, therefore, percentage of damaged pods for CW variety is zero.


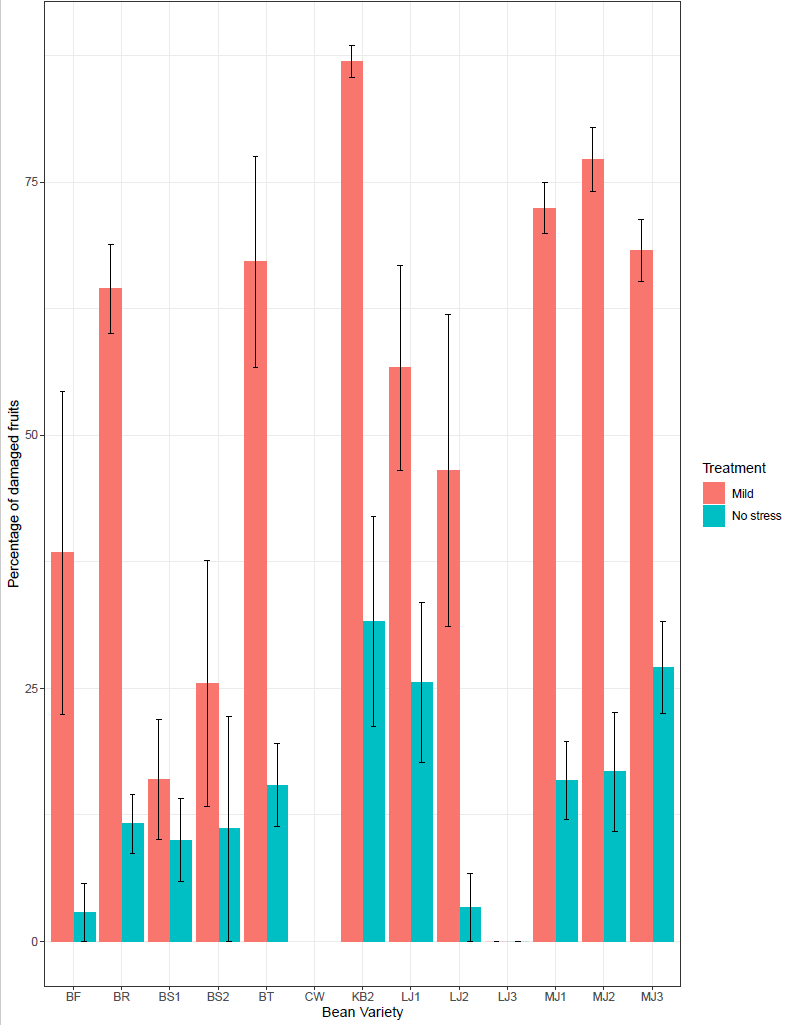


*Figure 2: Percentage of damaged pods by week 8 across bean varieties under different water stress treatments. Mild treatment bar implies drought stress.*

**Plant biomass:** Overall plants with drought stress had lower biomass than plants without stress (Figure S3). Amongst no stress plants, CW variety had the highest biomass and LJ1 variety the lowest. Amongst plants with drought stress, LJ2 variety had the highest biomass and LJ1 the lowest. The trend in biomass variation did not follow or contrast the trend in yield variation across varieties, i.e. plants with highest yield did not have highest or lowest biomass.


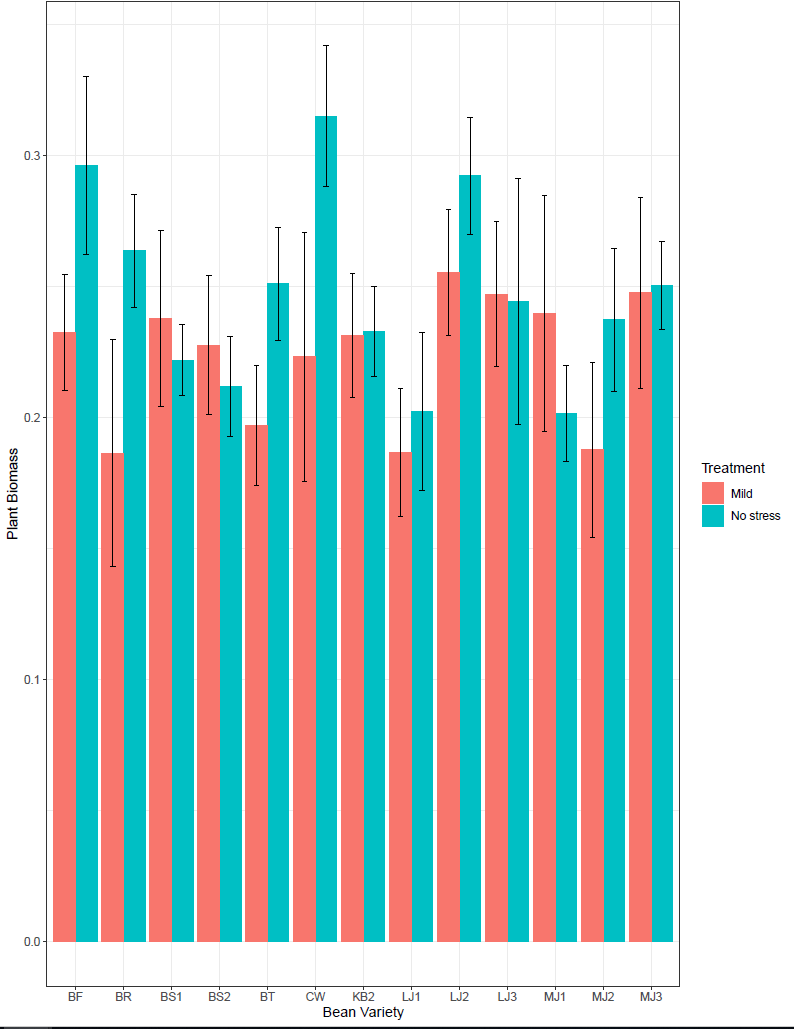


*Figure 3: Variation in plant biomass across bean varieties under two water stress treatments. Mild treatment bar implies drought stress.*

**Quantum Yield:** Overall QY was lower in drought stress plants (0.58) than in plants without water stress (0.66). For drought stress plants, QY was lowest for the BR variety (0.45) and highest for LJ3 variety (0.71) plants. Amongst ‘no stress’ plants, we did not record a large variation across varieties except for MJ3 (0.50) and BR (0.59) varieties that had the lowest QY values.

### Stress Indices

We used pod weight per plant to estimate different stress susceptible and stress tolerant indices. For selection criterion, varieties are considered tolerant when they show a higher value for MP, GMP, YSI, YI, and STI indices, whereas, for SSI they should show a lower value. We ranked varieties for all indices, where the most tolerant varieties were given the rank ‘one’. A cumulative score was calculated for each variety based on their rank on each index. Variety with the lowest score was considered the most tolerant variety; in our experiment, this was the BS1 variety. CW was found to be the least tolerant variety (Table S3).

Table 3: Scores of varieties on the different indices

| **Bean variety** | **Yp** | **Ys** | **MP** | **GMP** | **SSI** | **STI** | **YI** | **YSI** | **Cumulative score** |
| --- | --- | --- | --- | --- | --- | --- | --- | --- | --- |
| *BF* | *6* | *2* | *2* | *2* | *1* | *2* | *2* | *1* | *18* |
| BR | 5 | 7 | 8 | 7 | 7 | 7 | 7 | 7 | 55 |
| *BS1* | *4* | *1* | *1* | *1* | *2* | *1* | *1* | *2* | *13* |
| BS2 | 11 | 8 | 10 | 10 | 5 | 10 | 8 | 5 | 67 |
| BT | 9 | 4 | 7 | 5 | 4 | 5 | 4 | 4 | 42 |
| CW | 13 | 13 | 13 | 13 | 13 | 13 | 13 | 13 | 104 |
| KB | 10 | 11 | 11 | 11 | 9 | 11 | 11 | 9 | 83 |
| MJ1 | 8 | 5 | 9 | 6 | 6 | 6 | 5 | 6 | 51 |
| MJ2 | 2 | 6 | 3 | 4 | 8 | 4 | 6 | 8 | 41 |
| MJ3 | 7 | 3 | 4 | 3 | 3 | 3 | 3 | 3 | 29 |
| LJ1 | 1 | 10 | 5 | 8 | 12 | 8 | 10 | 12 | 66 |
| LJ2 | 3 | 9 | 6 | 9 | 11 | 9 | 9 | 11 | 67 |
| LJ3 | 12 | 12 | 12 | 12 | 10 | 12 | 12 | 10 | 92 |

Based on our calculated indices and other variables, we found varieties BS1, BF, MJ3, MJ2 and BT to be least negatively affected by drought stress. We did not have sufficient seeds for MJ3 and MJ2 variety as these were locally sourced from farmers in North Macedonia and the farmers only use these varieties for self-consumption. Therefore, we have selected varieties BS1, BF and BT to be used for our second plant diversity experiment.

## References

1. Papathanasiou F, Dordas C, Gekas F, Pankou C, Ninou E, Mylonas I, et al. The Use of Stress Tolerance Indices for the Selection of Tolerant Inbred Lines and their Correspondent Hybrids under Normal and Water-stress Conditions. Procedia Environ Sci. 2015 Jan 1;29:274–5.

2. A. RA, Hamblin J. Theoretical Aspects of Selection for Yield in Stress and Non-Stress Environment. Crop Sci. 1981;21(6):943–6.

3. Alefsi David Sánchez-Reinoso, Gustavo Adolfo Ligarreto-Moreno HR-D. Evaluation of drought indices to identify tolerant genotypes in common bean. J Integr Agric. 2019;
