# Supplementary material for: Effect of Drought on Bean Yield Is Mediated by Intraspecific Variation in Crop Mixtures
Source: Front Plant Sci. 2022 Jan 27;13:813417. doi: 10.3389/fpls.2022.813417 (PMC8829134; doi:10.3389/fpls.2022.813417)
Supplement: Supplementary file 2 [file Table_2.docx]

# Supplementary File 2

# Effect of drought on bean yield is mediated by intraspecific variation in crop mixtures

**Akanksha Singh^*^, Inea Lehner, Christian Schöb**

Agricultural Ecology Group, Institute of Agricultural Sciences, ETH Zurich, Zurich, Switzerland

***Correspondence**

Akanksha Singh

email: akanksha.singh@usys.ethz.ch

*Table S1: Effect of mixture type (****Beans*** *{monoculture/bean cultivar mixture/crop mixture};* ***companion plants*** *{monoculture/crop mixture)} and water treatment on total yield (bean seed number) and biomass (chickpeas, sorghum and sunflower) of all species*

|  | **Mixture type** | | | | **Water Treatment (Drought stress/No water stress)** | | | |
| --- | --- | --- | --- | --- | --- | --- | --- | --- |
| **Response variable (per pot)** | *Mean Sq* | *NumDF, DenDF* | *F value* | *P value* | *Mean Sq* | *NumDF, DenDF* | *F value* | *P value* |
| *Total bean seed number* | 0.45 | 2, 14.97 | 3.87 | 0.044 | 60.74 | 1, 238.18 | 518.91 | <0.001 |
| *Total sorghum biomass* | 11.25 | 1, 4.85 | 33.58 | 0.002 | 6.45 | 1, 88.75 | 19.25 | <0.001 |
| *Total chickpea biomass* | 4.01 | 1,4.85 | 10.59 | 0.023 | 2.44 | 1, 99.16 | 6.45 | 0.012 |
| *Total sunflower biomass* | 2.20 | 1, 4.65 | 23.48 | 0.006 | 18.82 | 1, 90.27 | 200.63 | <0.001 |

*Table S2: Average variation in bean seed number and biomass of companion plant species in different mixture types and water treatments*

|  | **Total productivity of different plant species per pot** | | | |
| --- | --- | --- | --- | --- |
|  | *Bean seed number* | *Sorghum biomass (grams)* | *Chickpea biomass (grams)* | *Sunflower biomass (grams)* |
| **Mixture Type** |  | | | |
| *Crop* | 8.51±0.08 | 2.38±0.05 | 3.29±0.06 | 11.3±0.17 |
| *Monoculture* | 15.6±0.09 | 16.9±0.23 | 8.32±0.10 | 20.1±0.24 |
| *Bean Cultivar* | 15.1±0.09 | NA | NA | NA |
| **Water Treatment** |  | | | |
| *Drought Stress* | 6.16±0.03 | 3.71±0.17 | 3.35±0.06 | 7.85±0.13 |
| *No Water Stress* | 15.5±0.06 | 5.54±0.19 | 4.77±0.10 | 17.6±0.17 |


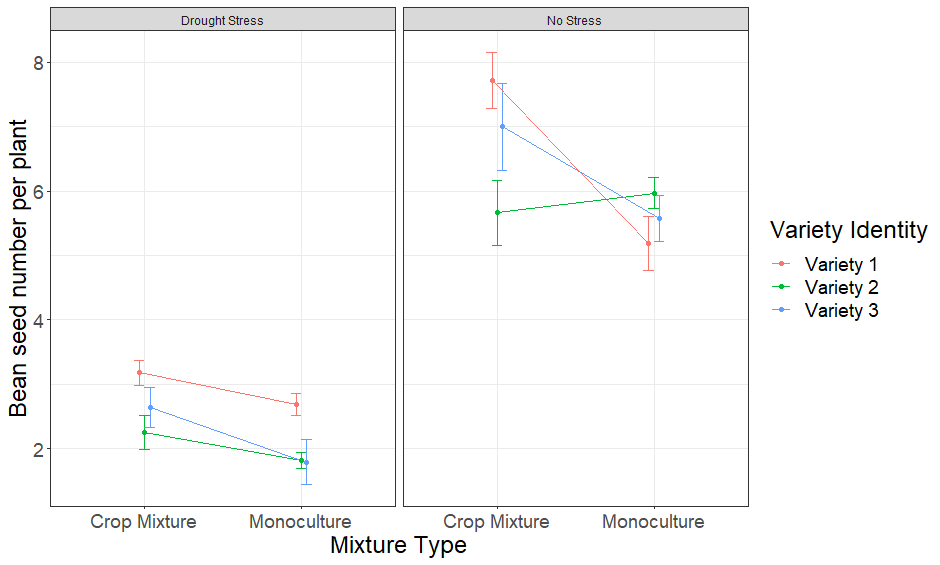


*Figure S1(a): Effect of mixture type and variety identity on bean yield per plant, with and without drought stress*


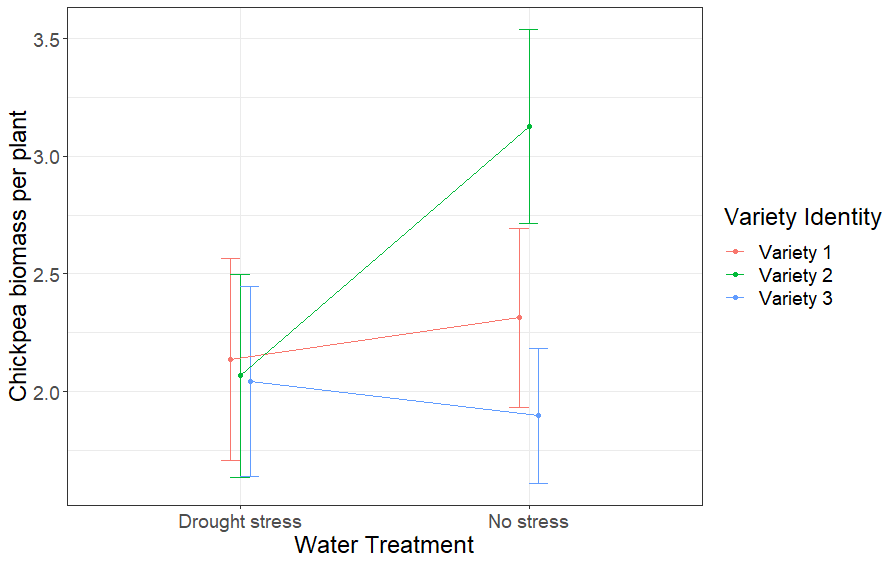

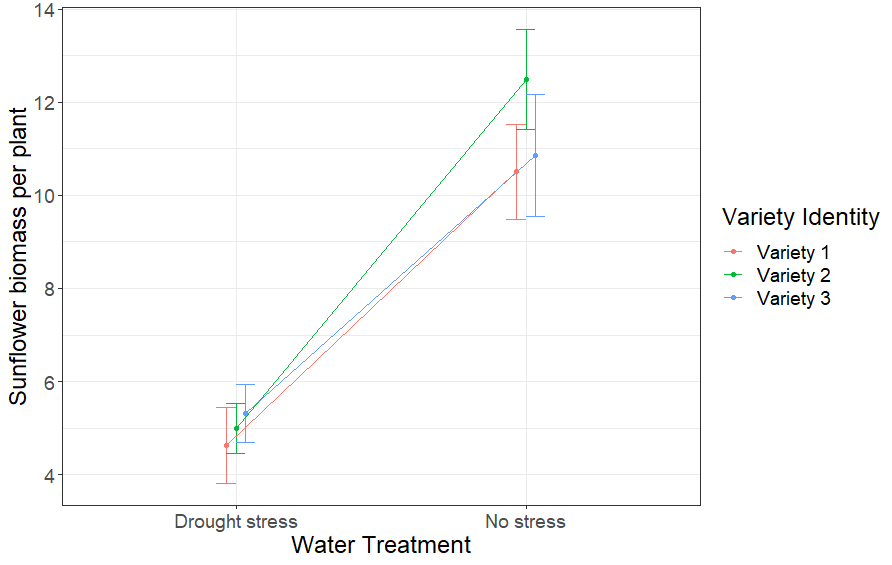


*Figure 1(b)* *Figure 1(c)*

*Figure S1: Effect of variety identity and drought stress on (b) chickpea and (c) sunflower biomass per plant.*


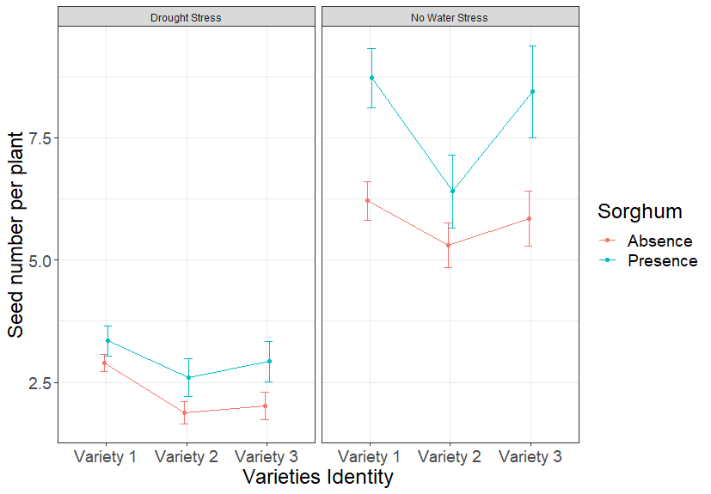


*Figure S2: Effect of variety identity on bean yield in the presence and absence of sorghum, under two water treatments (single variety pots).*


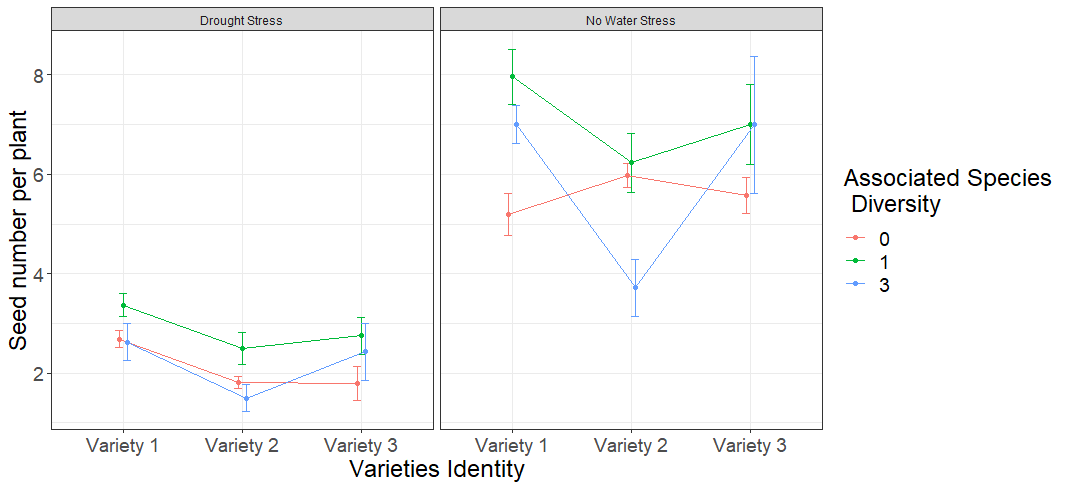


*Figure S3: Effect of interaction between variety identity and associated species diversity on average bean yield across different water treatments (single variety pots). Error bars represent ±1 SE.*

*Table S3: Effect of variety identity and companion plant species identity on leaf number of beans at week 6 in single bean variety pots*

| **Explanatory variables** | **Mean sq** | **numDF** | **denDF** | **F-value** | **P-value** |
| --- | --- | --- | --- | --- | --- |
| Varieties Identity | 0.827 | 2 | 205.00 | 32.405 | <0.001 |
| Sorghum P/A | 0.624 | 1 | 205.49 | 24.428 | 0.005 |
| Sunflower P/A | 8.455 | 1 | 205.44 | 331.033 | <0.001 |
| Chickpeas P/A | 1.788 | 1 | 205.53 | 70.016 | <0.001 |
| Varieties Identity*Sunflower | 0.118 | 2 | 205.00 | 4.611 | 0.011 |

*
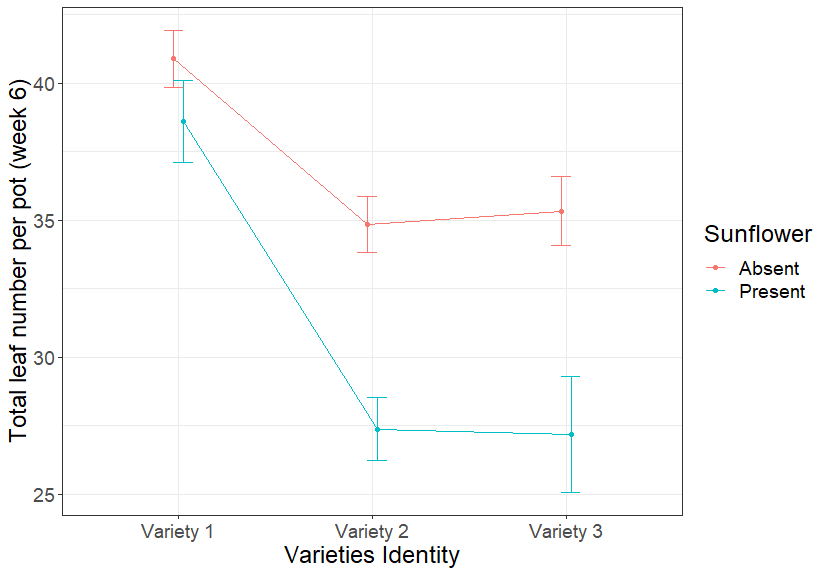
*

*Figure S4: Effect of variety identity on leaf number (week 6) in the presence and absence of sunflower (single variety pots). Error bars represent ±SE.*
